# Alleviating Distortion and Improving the Young’s Modulus in Two-Photon Polymerization Fabrications

**DOI:** 10.3390/mi9120615

**Published:** 2018-11-22

**Authors:** Chow-Shing Shin, Tzu-Jui Li, Chih-Lang Lin

**Affiliations:** 1 Department of Mechanical Engineering, National Taiwan University, Taipei 10617, Taiwan; csshin@ntu.edu.tw (C.-S.S.); r02522535@ntu.edu.tw (T.-J.L.); 2 Graduate Institute of Biotechnology and Biomedical Engineering, Central Taiwan University of Science and Technology, Taichung 40601, Taiwan

**Keywords:** two-photon polymerization, young’s modulus, inter-voxel distance, post-fabricated curing

## Abstract

Two-photon polymerization enables the extremely high resolution three-dimensional printing of micro-structures. To know the mechanical properties, and better still, to be able to adjust them is of paramount importance to ensuring the proper structural integrity of the printed products. In this work, the Young’s modulus is measured on two-photon polymerized micro-cantilever bars. Optimizing the scanning trajectory of the laser focus points is important in alleviating distortion of the printed bars. By increasing the laser power and decreasing the inter-voxel distances we can double the Young’s modulus. Post-curing with ultraviolet light can approximately quadruple the Young’s modulus. However, the resulting modulus is still only about 0.3% of that of the bulk polymerized material.

## 1. Introduction

As one of the mechanisms employed in 3D printing, photopolymerization is particularly attractive due to its flexibility in tailoring material properties. As the raw material it uses is liquid resin, the properties of the resulting printed structure can easily be modulated by adding other ingredients. For example, magnetic [1,2,3,4] or conductive [5,6,7] particles may be added to the resin to make the resulting structure ferromagnetic or electrically conductive. Moreover, reinforcing agents may be added to produce a composite material. Photopolymerization may be induced by the single-photon or two-photon processes. In the former, polymerization is initiated by relatively high energy ultraviolet (UV) or near UV photons [8]. Since these high energy photons will be intercepted on entrance to the resin, polymerization essentially occurs only in the surface layers to form a 2D layer. 3D structures are obtained by stacking together a number of these 2D layers. When the light intensity is extremely high, two relatively low energy photons may be simultaneously absorbed to emit a high energy photon which can bring about polymerization. Such high-intensity light is often produced by converging a laser beam to a fine focus. The beam itself will not induce polymerization and two-photon polymerization (TPP) can only occur in a minute micron or sub-micron voxel inside the beam focus where the energy density is intense enough. By moving the focus in a three-dimensional space in the resin, true 3D structures with submicron dimensional resolution may be fabricated [9].

Currently most of the works on TPP have been dedicated to developing the technique [10,11,12,13] and applying the technique to fabricate complex shaped microstructures [14,15,16], micro-machineries [17,18,19,20,21,22,23,24,25], micro/opto/electro-fluidic systems [26,27], and scaffolds for tissue engineering [28,29]. It has been demonstrated that a proper scaffold design and material stiffness can lead mesenchymal stem cells without osteogenic stimulation to differentiate towards osteogenic lineage [29]. Knowledge on the stiffness of the material and the ability to control it is a prerequisite to designing the TPP structures with reasonable integrity. Miwa [30] used a focussed UV laser beam with single photon polymerization to fabricate a sub-millimeter sized cantilever bar and measured a Young’s modulus of ~660–1080 MPa. These are of a typical order of magnitude similar to that of the bulk polymerized material. On the other hand, the Young’s modulus measured on TPP material [31,32,33,34,35] is often found to be smaller than that of the bulk material. Some of these are of the same order of magnitude but a fraction of the bulk modulus [32,34]. Others found the modulus of the TPP material several orders of magnitude smaller than the bulk value [33,35]. In both cases, post-curing with UV light improved the modulus further. However, study on the strategy of TPP fabrication for alleviating distortion and improving the Young’s modulus is still limited. This work aims at evaluating the effect of TPP fabricating parameters such as laser power, inter-voxel distance and laser scanning trajectory on the resulting TPP micro-structures.

## 2. Materials and Methods

**Figure 1:**
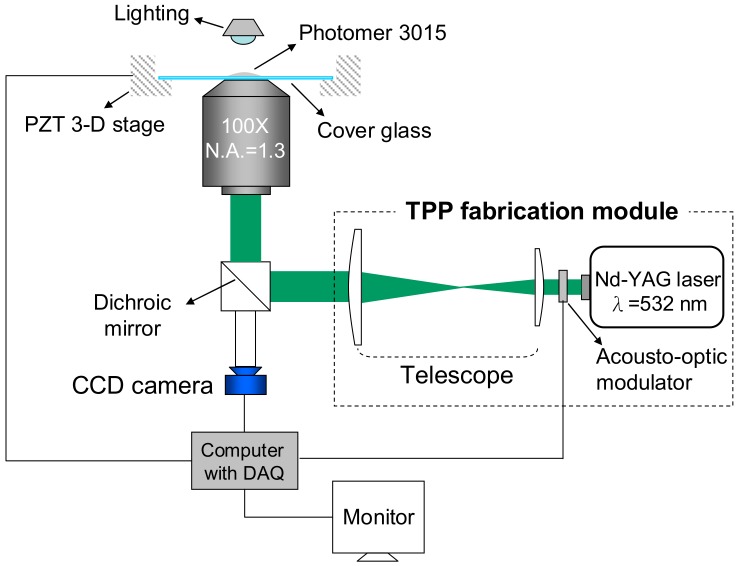
Schematic set up for two-photon polymerization (TPP) fabrication: The commercial resin (Photomer 3015) with 0.3% of photo-initiator is placed on a cover slip. The TPP fabrication module generates laser pulses of duration 0.55 ns at a 12 kHz repetition rate from a 532 nm Nd:YAG microchip laser. The laser energy was focused into the resin using an oil immersion 100× objective microscope. The cover slip is moved with a nano-positioning stage to allow the focal point to traverse any designated trajectory.

A commercial bisphenol A epoxy diacrylate resin (Photomer 3015, IGM Resins, Taoyuan, Taiwan) with 0.3% of *N*,*N*,*N*′,*N*′-Tetrakis (4-methoxyphenyl) benzidine (Sigma-Aldrich, St. Louis, MO, USA) added as a photo-initiator was placed on a cover slip. A laser pulse of duration 0.55 ns at a 12 kHz repetition rate from a 532 nm Nd:YAG microchip laser (Teem Photonics, Edinburgh, UK) was focused into the resin using an oil immersion 100× objective microscope (Olympus UPlanFLN, Olympus Co., Tokyo, Japan). The average and peak powers of the laser were 25 mW and 3.5 kW, respectively. The cover slip was moved with a nano-positioning stage (P-611.3 NanoCube^®^ XYZ Piezo Stage, Physik Instrumente, Karlsruhe, Germany) to allow the focal point to traverse any designated trajectory. The fabrication set-up is shown schematically in Figure 1.

**Figure 2:**
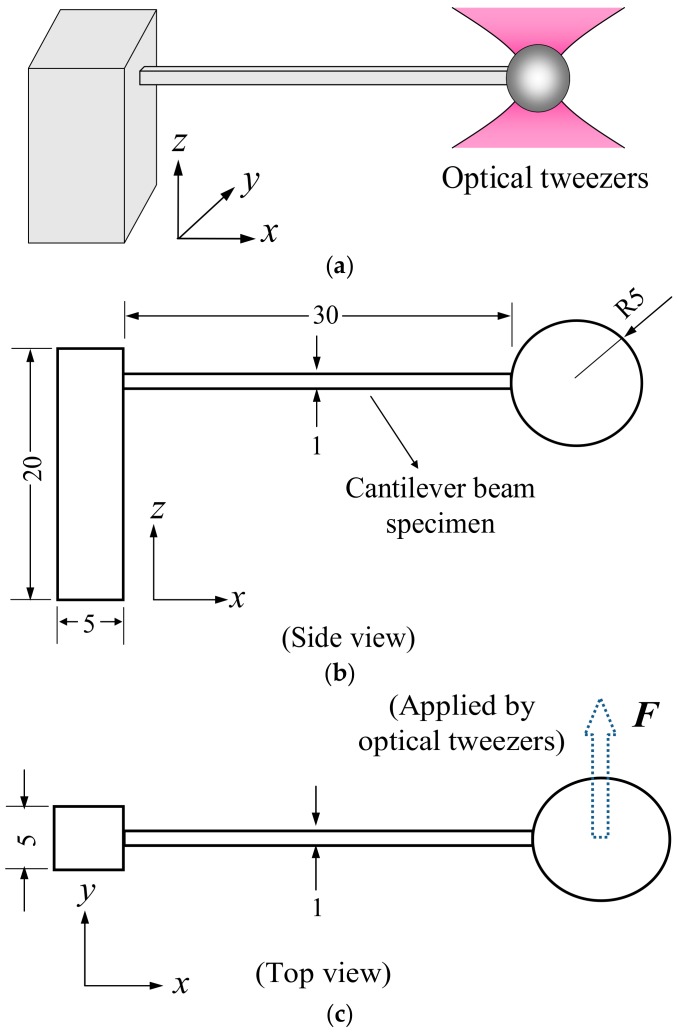
(**a**) Overall design of the specimen; (**b**) Side view; (**c**) Top view (dimensions in μm). A 5 μm diameter sphere was fabricated at the tip of the bar for facilitating force application. By dragging the sphere sideways using optical tweezers, a horizontal force is applied to bend the cantilever bar.

To measure the Young’s modulus, cantilever bar specimens with nominal dimensions 1 μm × 1 μm × 30 μm were fabricated. The bar was hung out from a relatively rigid pillar which was attached to the cover slip. Since the force needed to bend the specimen is of the order of a piconewton, optical tweezers (Zeiss PALM MicroTweezers, Oberkochen, Germany) with a maximum laser power output of 135.85 mW were employed. The mechanism of optical tweezers has been described in previous works [36,37] and its ability to apply an accurate force on a sphere has been demonstrated [38,39,40] and used for the measurement of mechanical properties [17,23,33,35]. The manipulation of the optical tweezers was presented in previous work [17,23]. To facilitate force application, a 5 μm diameter sphere was fabricated at the tip of the bar. The overall design and dimensions of the specimen are shown in Figure 2. By dragging the sphere sideways, a horizontal force is applied to bend the cantilever bar as shown in Figure 2c. The deflection at the tip of the bar was recorded when the sphere had just escaped from optical trapping [17]. Calibration indicated the maximum power corresponded to a force of 161 pN on the sphere.

**Figure 3:**
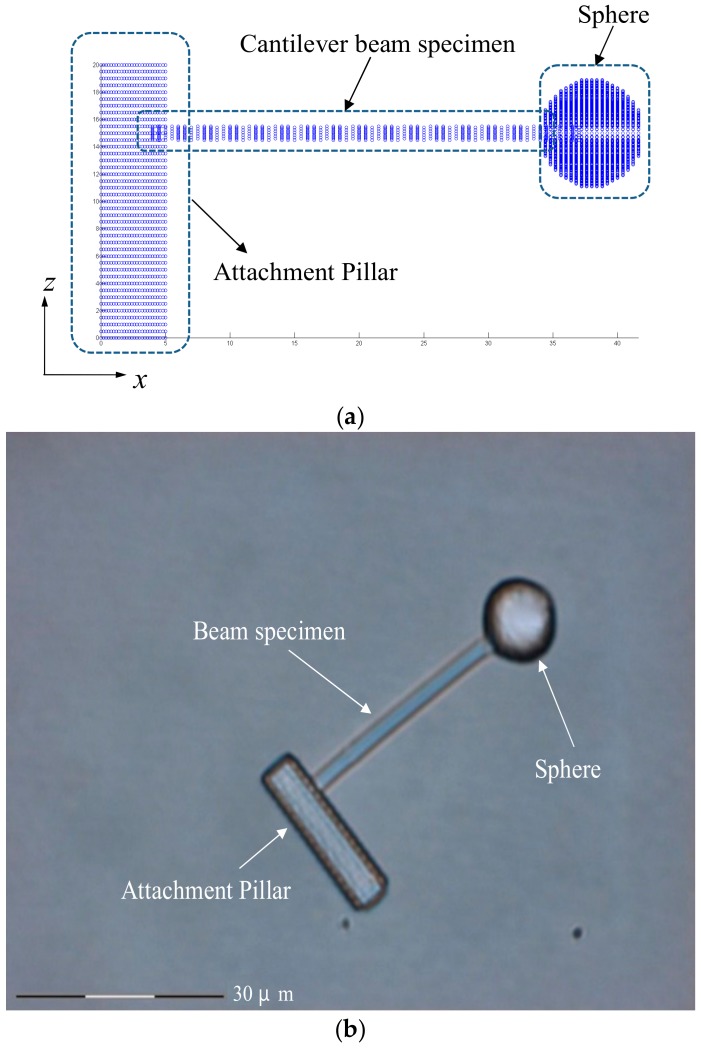
(**a**) The slicing scheme for TPP fabrication; (**b**) The image of a resulting specimen which has been made to fall flat on the cover slip through a purposely weakened foundation of the attachment base.

The slicing scheme showing progressive planes for fabrication is depicted in Figure 3. The naming of the *x*, *y* and *z* axes shown in the coordinate systems in Figure 2 and Figure 3 will be used throughout this work. An image of the specimen which has been made to fall flat on the cover slip through a purposely weakened foundation of the attachment base is also shown in Figure 3. After fabrication, the cover slip is dipped gently into alcohol to wash away the uncured resin. With respect to the small sectioned bar, the sphere is relatively massive and will cause bending in the bar. To counter this effect of gravity, as well as to facilitate the application of optical tweezers, the specimens were buffered in alcohol during tests.

**Figure 4:**
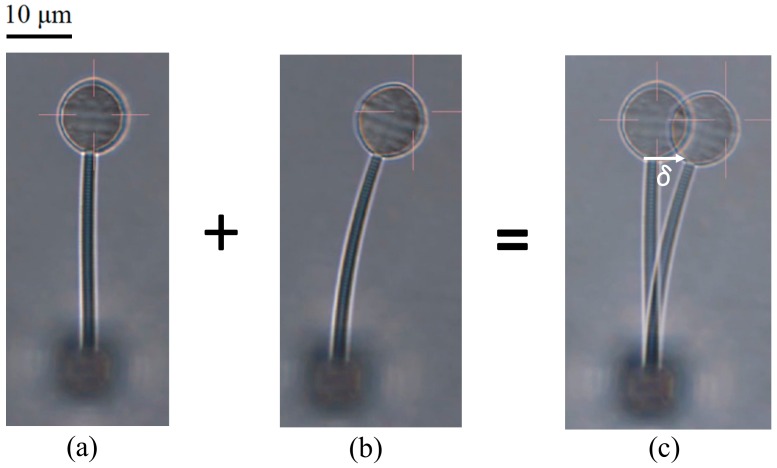
Typical photomicrographs of a bar specimen: (**a**) Before bending; (**b**) during bending and (**c**) superimposed images to measure the deflection *δ*.

Photomicrographs of the bar before and during the application of bending force were recorded by the microscope of the optical tweezers and the two images were superimposed to give the deflection *δ* at the tip of the bar (Figure 4). The force *F* acting on the center of the sphere (Figure 2) may be transformed into an equivalent system of a force *F* and a couple *M* (=*Fr*) acting on the tip of the bar [41]. The deflection *δ* by this force *F* and couple *M* is shown in Reference [42]:

(1) $$\delta =\frac{FL^{3}}{3EI}+\frac{ML^{2}}{2EI}$$

where *L* is the length of the bar, *r* is the radius of the tip sphere, *E* is the Young’s modulus and *I* is the second moment of area of the bar section.

Thus, the Young’s modulus *E* can be computed by:

(2) $$E=\frac{FL^{2}}{6\delta I}(2L+3r)$$

**Figure 5:**
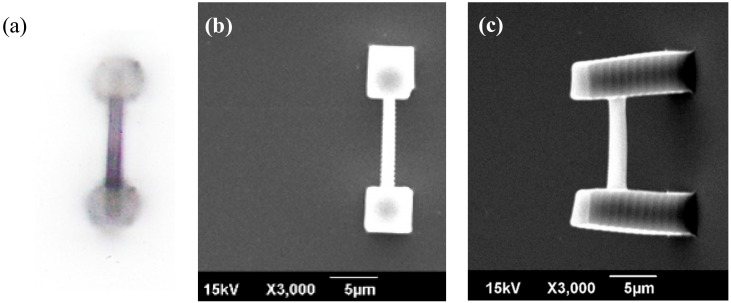
(**a**) Optical microscope normal incidence; (**b**) SEM normal incidence and (**c**) SEM 45° perspective views of a typical double pillar bar. The normal view allows the width of the bar to be measured and the 45° perspective give estimates of the depth of the bar.

Optical micrographs such as that in Figure 4 allow the bar width to be measured but the diffraction effect around the specimen edges will give an overestimation of the actual dimensions. The specimen edges are much sharper when observed with a scanning electron microscope (SEM) and should provide a more accurate measurement. However, without the buffering alcohol, the cantilever bar deflects and deforms under gravity and this will affect the accuracy of measurement. To alleviate errors in the measured bar dimensions, measurements were first made under the optical microscope. Another set of bars (fabricated using exactly the same parameter combinations but with the sphere replaced by another rigid pillar) were measured both under the optical microscope and by the SEM. The ratio between the SEM and optical microscope values was used to correct for the cantilever bar values. Moreover, the depth of the bar was inferred from a 45° view in the SEM. Errors in bar depth are expected to be higher due to this indirect method but since the bars will be bent in the *x*-*y* plane, error in depth affects *I* and *E* to the first degree. Figure 5 shows the normal incidence views by the optical microscope and the SEM as well as a 45° perspective view of a typical double pillar bar.

To study the effect of the fabricating laser power, the maximum and minimum powers that can give successful fabrication were first evaluated through trial and error. Four different power levels within this range were then chosen while the inter-voxel distances were kept constant as listed in Table 1. On the other hand, the effect of the inter-voxel distance was evaluated with a fixed laser power of 0.15 mW while the inter-voxel distances on the *x*-*y* plane and in the *z* direction are varied as listed in Table 2. The effect of post-fabrication curing by ultraviolet (UV) illumination has also been studied using a batch of cantilever bar specimens fabricated with the case 1-2 conditions in Table 1 and irradiated with a 4 W 365 nm UV lamp (UVGL-25compact UV lamp, UVP, LLC, Upland, CA, USA) for different lengths of time. In each of the above fabrication conditions, three samples were measured. The average values are plotted with the standard deviations indicated on error bars in the following figures. For comparison, a bulk specimen that was 4 mm thick, 10 mm wide and 120 mm long was also tested by bending to obtain its Young’s modulus. The bulk specimen was prepared using the same resin but because of its thickness curing with the above UV lamp took 48 h.

## 3. Results and Discussion

### 3.1. Effect of Rastering Pattern on the Specimen Shape

**Figure 6:**
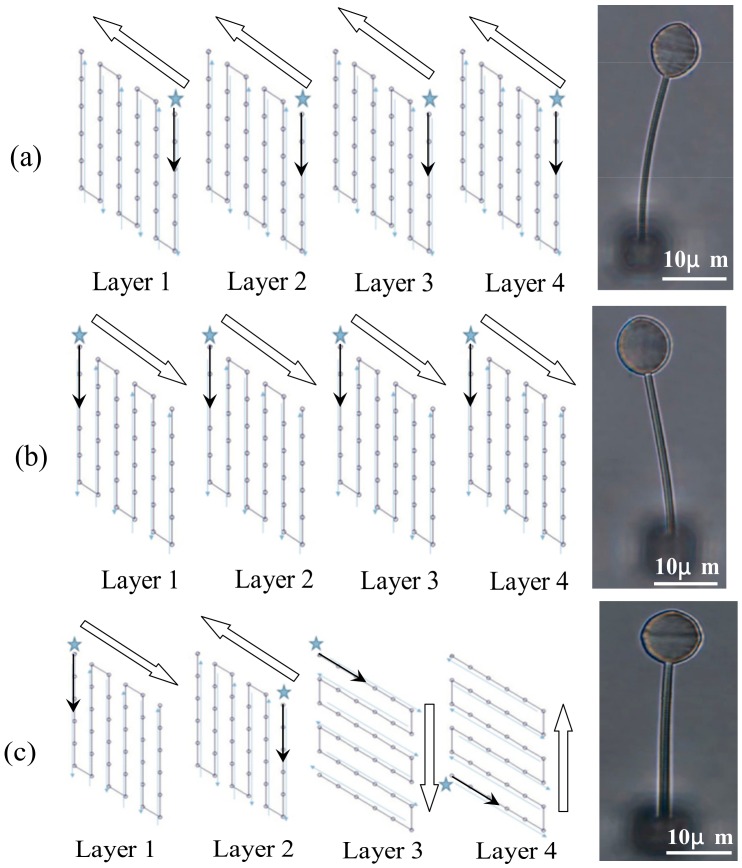
Different rastering directions (stars indicate the starting point of a path) and the top view of the resulting specimens: When the trajectories start at the same corner and follow the same direction in each plane, (**a**,**b**) the resulting bar will curve to the side of the trajectory starting point and the sphere is elongated; when the rastering directions in adjacent planes are alternated, (**c**) a straight bar with a relatively undistorted sphere resulted.

TPP basically cures the resin on a point by point scheme. The slicing in Figure 3a shows planes in the structure. The laser focus has to traverse a rastering trajectory in each plane to construct that plane. When the trajectories start at the same corner and follow the same direction in each plane, the resulting bar will curve to the side of the trajectory starting point. This occurred in every specimen fabricated and Figure 6a,b shows two typical results.

The spheres at the tip of the bars are also slightly distorted in shape. Volume changes will occur during polymerization and progressive curing along a trajectory may give rise to a residual stress pattern that may account for this initial curvature of the bar. When the rastering directions in adjacent planes are alternated as shown in Figure 6c, a straight bar with a relatively undistorted sphere resulted and this method was adopted in the current work. The above phenomenon suggests that the rastering path design in any TPP fabrication should try to balance the effect of residual stress in order to alleviate distortion in the final product.

### 3.2. Effect of Laser Power

#### 3.2.1. Effect of Laser Power on the Dimensions of the Fabricated Product

**Figure 7:**
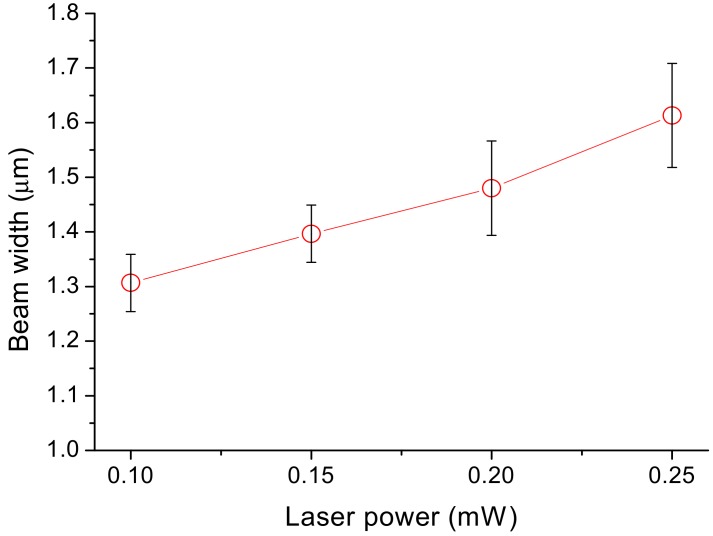
Effect of laser power on the cantilever bar width: The variation in the width of the cantilever bars with laser power under fixed inter-voxel *x* and *y* distances of 0.13 ìm and an inter-voxel *z* distance of 0.16 ìm. The circles represent data averaged from three specimens and the error bars indicate the standard deviation of data. As the average laser power increases from 0.1 to 0.25 mW, the width increases from 1.31 to 1.61 ìm.

Figure 7 shows the variation in the width of the cantilever bars with respect to laser power under fixed inter-voxel *x* and *y* distances of 0.13 μm and an inter-voxel *z* distance of 0.16 μm. In all cases, the dimensions were larger than the designated 1 μm. As the average laser power increases from 0.1 to 0.25 mW, the width increases from 1.31 to 1.61 μm. A one-way analysis of variance (ANOVA) with Bonferroni’s post-hoc test indicated that the differences between adjacent laser power groups were insignificant but between the 0.1 and 0.25 mW groups there was p value of 0.02, which indicates a significant difference between these two laser powers. The designated bar cross-section of 1 μm × 1 μm limits the trajectory of the center of the laser focus. Around the focus there is a certain ellipsoidal region called the voxel where the light intensity is high enough to bring about TPP. This explains why the actual dimensions are always larger than in the design. With higher laser energy, the curable voxel region is larger, giving rise to the observed increase in dimensions with respect to laser power.

#### 3.2.2. Effect of Laser Power on the Young’s Modulus of the Fabricated Product

**Figure 8:**
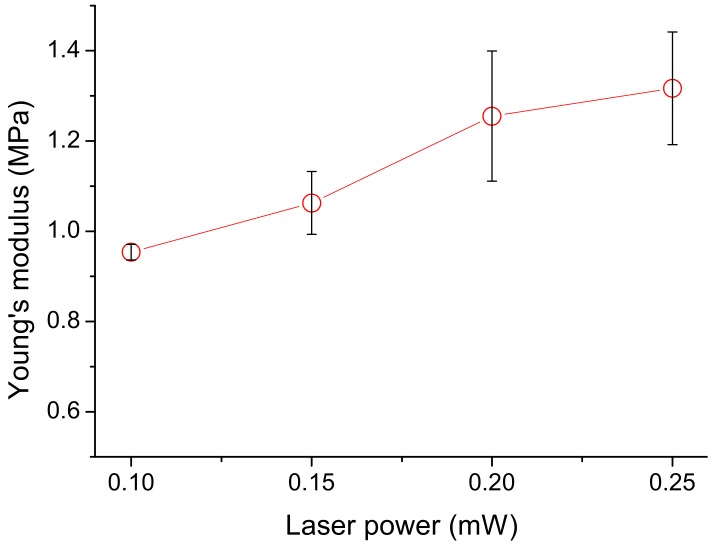
Variation of Young’s Modulus with laser power: Increasing the average laser power from 0.1 to 0.25 mW increased the Young’s modulus from ~965 kPa to ~1750 kPa. The circles represent data averaged from three specimens and the error bars indicate the standard deviation of data.

Increasing the average laser power from 0.1 to 0.25 mW increased the Young’s modulus from ~965 kPa to ~1750 kPa (Figure 8). With power higher than 0.25 mW, occasional localized overheating occurs which leads to evaporation and the formation of large bubbles which disrupt fabrication. Again, one-way ANOVA with Bonferroni’s post-hoc test indicated that the results between the 0.1 and 0.25 mW groups exhibited significant differences (*p* = 0.04). Among other power groups the *p* values are greater than 0.05. Increasing the laser power will increase the light intensity within a voxel as well as increasing its size. The former may promote the degree of polymerization and cross-linking. The packing of ellipsoidal voxels inevitably leaves some unfilled cavities between adjacent voxels. Resin trapped in these cavities will remain uncured and does not contribute towards the Young’s modulus. Increasing the voxel size will lead to more overlapping between adjacent voxels under a fixed inter-voxel distance, thus decreasing the amount of uncured resin trapped between neighboring voxels. Both of the above effects help to improve the Young’s modulus.

### 3.3. Effect of Inter-Voxel Distances on the Young’s Modulus of the Fabricated Product

**Figure 9:**
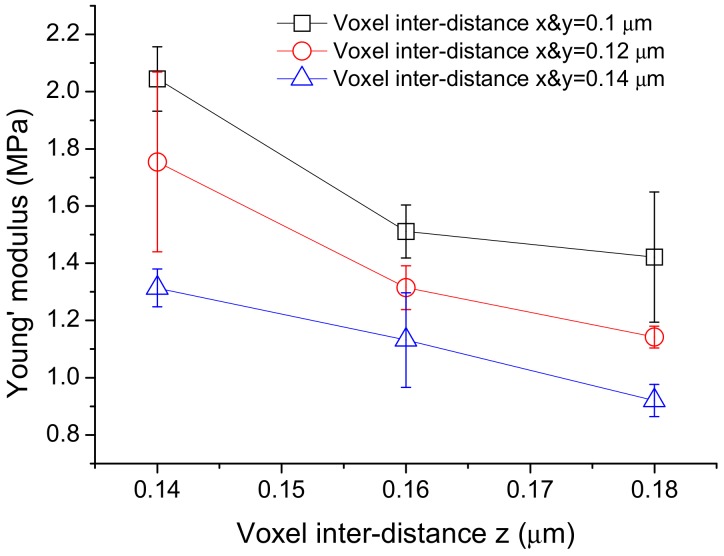
Variation of Young’s modulus with inter-voxel distances: Young’s modulus from cantilever bars with the same nominal dimensions that were fabricated with an average laser power of 0.15 mW but with different inter-voxel distances. Each data point is averaged from three specimens and the error bars indicate the standard deviation of data. In all cases, the average Young’s modulus increases with a decreasing inter-voxel distance.

The Young’s moduli from cantilever bars with the same nominal dimensions that were fabricated with an average laser power of 0.15 mW but with different inter-voxel distances are shown in Figure 9. In all cases, the average Young’s modulus increases with a decreasing inter-voxel distance. A one-way ANOVA with Bonferroni’s post-hoc test indicated that differences in the Young’s modulus resulting from a change of 0.04 μm in inter-voxel distance in either the *x* and *y* directions or the *z* direction were significant. Decreasing the distance by 0.04 μm in either the *x*, *y* or *z* direction increases the Young’s modulus by ~25–35%. Again, this may be attributed to the reduction in the amount of uncured resin as noted above. With a further decrease in the inter-voxel distance, fabrication is disrupted by the occasional localized formation of large bubbles.

### 3.4. Effect of Post-Fabricated UV Exposure

**Figure 10:**
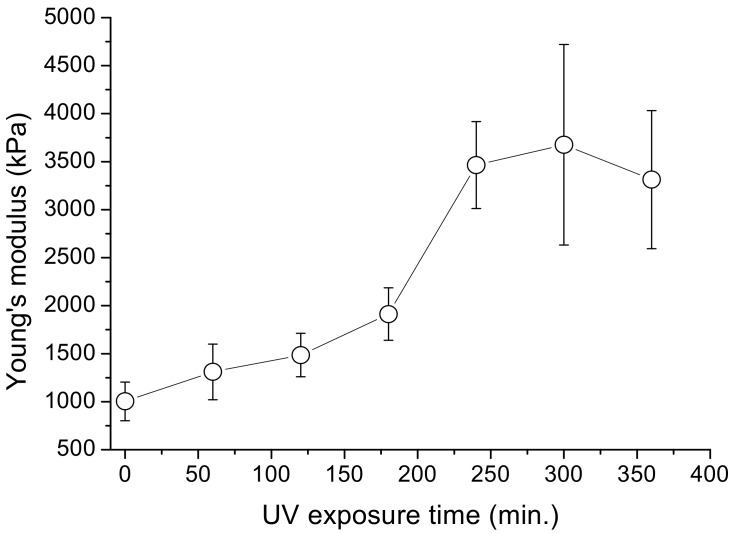
Variation of the Young’s modulus as a function of UV post-cure exposure time: Young’s modulus increases from ~1000 to ~3700 kPa within the first 300 min of post-fabrication UV exposure. The circles represent data averaged from three specimens and the error bars indicate the standard deviation of data.

Besides packing more voxels into a cantilever bar, the amount of uncured resin may also be reduced by irradiating the fabricated specimens with ultraviolet light (UV). Young’s modulus increases from ~1000 to ~3700 kPa within the first 300 min of UV exposure (Figure 10). This may be attributed to the curing of the trapped resin. With prolonged exposure, the Young’s modulus tends to drop. A marked increase in scatter can be seen for exposure times of 240 min and beyond. Incidentally, a one-way ANOVA with Bonferroni’s post-hoc test indicated that the differences in the Young’s moduli between those fabricated and those irradiated for 240 min and beyond were significant. Besides promoting polymerization, the high energy UV light may also break bonds and degrade polymers [43]. The increase in scatter can probably be attributed to these two effects competing with each other while the subsequent observed drop in the Young’s modulus may be associated the dominance of the latter effect.

The specifications of the Photomer 3015 provided by the resin supplier have not documented the Young’s modulus of the resin but listed a tensile strength of 7500 Psi and an elongation of 6% [44]. Assuming the resin remains linearly elastic until tensile failure, this gives a conservative estimate of a modulus of 860 MPa. The actual modulus will be higher if the resin exhibits some degree of plasticity under tension. The bulk UV cured specimen in the current work gives a modulus of 1042 MPa, which corroborates well with that which was deduced from the supplier’s information. Thus, the modulus of the bulk cured material by direct UV is about 500–1100 times higher than that of the fabricated TPP material and 280 times that of the post-fabricated UV irradiated material. The large discrepancy between the bulk cured and TPP materials is unlikely to be because of the uncured resin trapped between the voxels alone. A more significant effect may be that the degree of polymerization and cross-linking is much more limited in TPP. This is understandable as curing is confined to the tiny volume of a voxel, where the components of the resin may quickly become stoichiometrically disproportionate, yet replenishment is difficult due to the high viscosity of the partially cured resin [32]. Raman microspectroscopy showed a considerable amount of carbon double bonds which need to be broken to allow for polymerization and cross-linkage. These bonds can remain unconsumed in TPP materials [33,45]. A three order of magnitude difference of the bulk modulus has also been reported in some TPP nanowire springs [33,35]. On the other hand, a slightly reduced but still of similar order modulus as the bulk materials has been reported in some TPP structures at the micrometer level [32,34]. Nakanishi et al. [33] suggested that this contrasting phenomenon may be a size effect as the glass transition temperature may drop significantly when the material thickness drops below 300 nm, where the glass transition can reduce the modulus by several orders of magnitude. The current work indicates that this size effect cannot be a major contribution as the cantilever involved is in the micrometer level. The different chemistries in different resin systems may play an important role in determining why some obtained similar moduli and why some moduli were significantly reduced. This aspect is worth further investigation and clarification.

The observation that the Young’s modulus can be controlled to some extent by modifying the laser power and inter-voxel distance opens up the possibility of manufacturing a functional material/structure with a designable stiffness gradient. It is interesting and worth looking into to see whether other material properties exhibit similar effects. The above results clearly point out that in designing a structure that will be fabricated through the TPP technique, one should bear in mind that for some resin systems, the Young’s modulus could be two to three orders of magnitude lower than that of the bulk polymerized material. To ensure a good stiffness, one should employ the highest allowable laser power as well as the most closely packed voxels. Post-fabricated UV irradiation can also be very effective, however there is an optimum exposure duration, beyond which the material stiffness will start to deteriorate.

## 4. Conclusions

This study evaluates the effect of TPP fabricating parameters such as laser power, inter-voxel distance and laser scanning trajectory on the resulting TPP micro-structures. Within the usable ranges of laser power and inter-voxel distance, the lowest Young’s modulus obtained in the TPP material from the Photomer 3015 resin was about 1000 kPa. This is about three orders of magnitude smaller than the bulk material modulus. A similar degree of difference between the bulk and TPP modulus has been reported. This may be due to the resin chemistry which is not favorable for efficient cross-linking in the tiny volume of a voxel. It may be worthwhile to design an experiment that varies the size of the voxels while keeping a similar power density to test the above hypothesis. Increasing the laser power can increase the modulus by ~100% while decreasing the inter-voxel distance can increase it by a maximum of ~35%. Post-fabrication UV irradiation can increase the modulus to ~3700 kPa. This does not only provides a means for improving the Young’s modulus but also opens up the possibility of manufacturing a functional material/structure with a designable stiffness gradient. When the dimensions of the structural components get smaller, the distortion due to inhomogeneous thermal and shrinkage effects may become more important. Our work shows that by designing the rastering trajectory of the laser focus to balance off the residual stress in successive layers, distortion can be alleviated. This study is expected to contribute to the applications of the TPP microfabrication for ensuring structural integrity.

## Figures and Tables

**Table 1:** Fabrication parameter combinations with different laser powers.

Cases	Power (mW)	Inter-Voxel *x* & *y* Distance (μm)	Inter-Voxel *z* Distance (μm)
1-1	0.10	0.13	0.16
1-2	0.15	0.13	0.16
1-3	0.20	0.13	0.16
1-4	0.25	0.13	0.16

**Table 2:** Fabrication parameter combinations with different inter-voxel distances.

Cases	Power (mW)	Inter-Voxel *x* & *y* Distance (μm)	Inter-Voxel *z* Distance (μm)
2-1	0.15	0.10	0.14
2-2	0.15	0.10	0.16
2-3	0.15	0.10	0.18
2-4	0.15	0.12	0.14
2-5	0.15	0.12	0.16
2-6	0.15	0.12	0.18
2-7	0.15	0.14	0.14
2-8	0.15	0.14	0.16
2-9	0.15	0.14	0.18
